# Molecular profiling reveals primary mesothelioma cell lines recapitulate human disease

**DOI:** 10.1038/cdd.2015.165

**Published:** 2016-02-19

**Authors:** T Chernova, X M Sun, I R Powley, S Galavotti, S Grosso, F A Murphy, G J Miles, L Cresswell, A V Antonov, J Bennett, A Nakas, D Dinsdale, K Cain, M Bushell, A E Willis, M MacFarlane

**Affiliations:** 1 MRC Toxicology Unit, University of Leicester, Leicester, UK; 2 UHL NHS Trust, Glenfield Hospital, Leicester, UK; 3 UHL NHS Trust, Leicester Royal Infirmary, Leicester, UK

## Abstract

Malignant mesothelioma (MM) is an aggressive, fatal tumor strongly associated with asbestos exposure. There is an urgent need to improve MM patient outcomes and this requires functionally validated pre-clinical models. Mesothelioma-derived cell lines provide an essential and relatively robust tool and remain among the most widely used systems for candidate drug evaluation. Although a number of cell lines are commercially available, a detailed comparison of these commercial lines with freshly derived primary tumor cells to validate their suitability as pre-clinical models is lacking. To address this, patient-derived primary mesothelioma cell lines were established and characterized using complementary multidisciplinary approaches and bioinformatic analysis. Clinical markers of mesothelioma, transcriptional and metabolic profiles, as well as the status of p53 and the tumor suppressor genes *CDKN2A* and *NF2*, were examined in primary cell lines and in two widely used commercial lines. Expression of MM-associated markers, as well as the status of *CDKN2A*, *NF2,* the ‘gatekeeper' in MM development, and their products demonstrated that primary cell lines are more representative of the tumor close to its native state and show a degree of molecular diversity, thus capturing the disease heterogeneity in a patient cohort. Molecular profiling revealed a significantly different transcriptome and marked metabolic shift towards a greater glycolytic phenotype in commercial compared with primary cell lines. Our results highlight that multiple, appropriately characterised, patient-derived tumor cell lines are required to enable concurrent evaluation of molecular profiles versus drug response. Furthermore, application of this approach to other difficult-to-treat tumors would generate improved cellular models for pre-clinical evaluation of novel targeted therapies.

Malignant mesothelioma (MM) is an aggressive, fatal tumor strongly associated with asbestos exposure. MM is responsible for ~3000 deaths per year in the United States and 5000 deaths in Western Europe.^1^ However, mortality rates are expected to increase by 5–10% year on year in most industrialized countries until about 2020,^2^ with the worldwide incidence predicted to plateau around 2030. In recent years the demography of MM has changed; the age of MM patients has decreased and there is an increased incidence in females, likely reflecting exposure from non-occupational sources.^3^ The major histologic subtypes of MM, epithelioid, sarcomatoid and biphasic are all associated with poor patient survival, with sarcomatoid MM exhibiting the worst prognosis.^4^ The median overall survival for MM following frontline chemotherapy with pemetrexed and cisplatin is ~12 months.^5^ The disease occurs after a long (up to 40 years) latency period and the delay between asbestos exposure and MM onset suggests that multiple factors are involved in asbestos-induced tumorigenesis. Moreover, the non-specific early symptoms combined with the older age of MM patients and the absence of reliable biomarkers hinders early diagnosis.

There is an urgent need to improve MM patient outcomes and this requires both appropriate pre-clinical models and new therapeutic strategies. Mesothelioma-derived cell lines are essential for the development of *in vitro* model systems, thereby enabling mechanistic studies of tumor pathogenesis, as well as the identification of new biomarkers and novel therapeutic targets. A number of commercially available cell lines have been widely used for translational *in vitro* studies.^6^ In addition, to date, several primary MM cell lines have been described, although their degree of characterization varies.^7, 8, 9, 10, 11, 12^ It is generally accepted that these primary cell lines are more physiologically relevant as *in vitro* models, although the generation of such lines is both challenging and labor intensive.

To evaluate *in vitro* models, a detailed comparison of long-established commercially available MM cell lines with freshly derived primary cell lines is essential. This is particularly important as MM is associated with chromosomal loss, deletions in *CDKN2A, CDKN2B*, and *NF2*^13, 14, 15^ genes and mutations in *BAP1* and *CUL1.*^16^ Thus, genomic instability limits the long-term usefulness of commercially available MM cell lines. To address this, and to develop more relevant pre-clinical models of MM, we established and characterised eight primary mesothelioma cell lines and employed a number of complementary multidisciplinary approaches to examine differences between these cell lines and two widely used commercial cell lines. Our data demonstrate that there is a significant difference between commercial and primary cell lines at the molecular level, including the transcriptome, expression of mesothelial markers and proteins associated with MM pathogenesis including p53, metabolic profile, status of the tumor suppressor genes *CDKN2A* and *NF2* and their products. Importantly, the commercial cell lines lack many key molecular features known to be associated with MM, whereas the eight primary cell lines more accurately recapitulate human disease, thus providing a superior model for pre-clinical evaluation of novel targeted therapies.

## Results

### Clinical specimens and establishment of primary cultures

MM is one of the most difficult cancers in terms of early diagnosis; as a result, tissue specimens representing early stages of MM are not generally available. Surgically resected tumor tissues were obtained from patients with advanced epithelioid (six cases) or biphasic (two cases) MM (Table 1), as surgery is not considered beneficial for patients with sarcomatoid MM. Occupational exposure to asbestos had been identified in five cases, whereas three patients had no known history of exposure. Primary mesothelial cultures were established by passaging the cells isolated from resected tissue and were characterized at low (<10) and high (30–50) passages.

### Morphological appearance of primary mesothelioma cell lines

**Figure 1:**
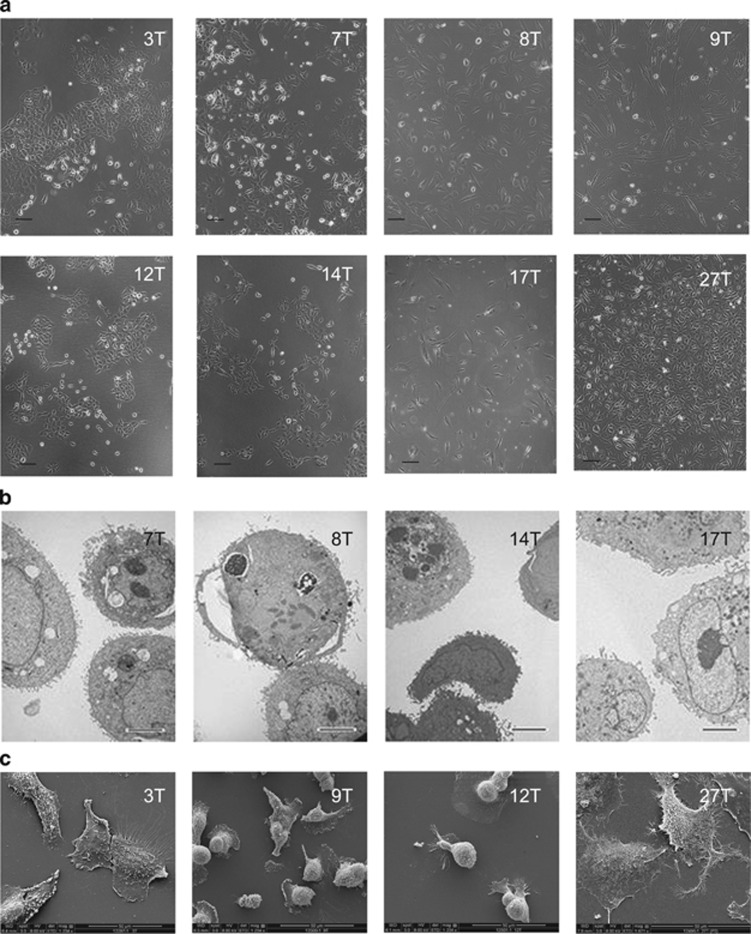
(**a**) Phase-contrast images of patient-derived cell lines, MESO-3T, MESO-7T, MESO-8T, MESO-9T, MESO-12T, MESO-14T, MESO-17T and MESO-27T, displaying mesothelial morphology, scale bar indicates 100 *μ*m. (**b**) Transmission electron microscopy and (**c**) scanning electron microscopy of primary mesothelioma cell lines showing characteristic microvilli on the cell surface. Scale bars indicate 5 and 50 *μ*m, respectively

Primary MM cell lines, including MESO-3T, MESO-7T, MESO-8T, MESO-9T, MESO-12T, MESO-14T, MESO-17T and MESO-27T, established in culture as an attached monolayer (Figure 1a and b). Adherent cells exhibited characteristic mesothelioid 'cobble-stone' morphology and were occasionally multi-nucleated or vacuolated. The cell lines were, to a certain extent, morphologically distinct and doubling times varied from 31.6 to 141.6 h (Supplementary Table 1). Long, thin, often branching microvilli on the cell surface, a characteristic feature of mesothelial cells, were observed in all primary cultures (Figure 1b and c). Primary tumor cells in culture formed tight intercellular junctions (Supplementary Figure S1), which are typically present in mesothelial cell cultures.^17^ Primary MM cell lines MESO-3T, MESO-7T, MESO-8T, MESO-12T, MESO-14T and MESO-27T exhibited the ability to grow to high passage (>30), whereas MESO-9T and MESO-17T could only be cultured for up to 15 passages before displaying signs of senescence.

### Expression of markers and proteins implicated in MM pathogenesis differs between primary and commercial cell lines

**Figure 2:**
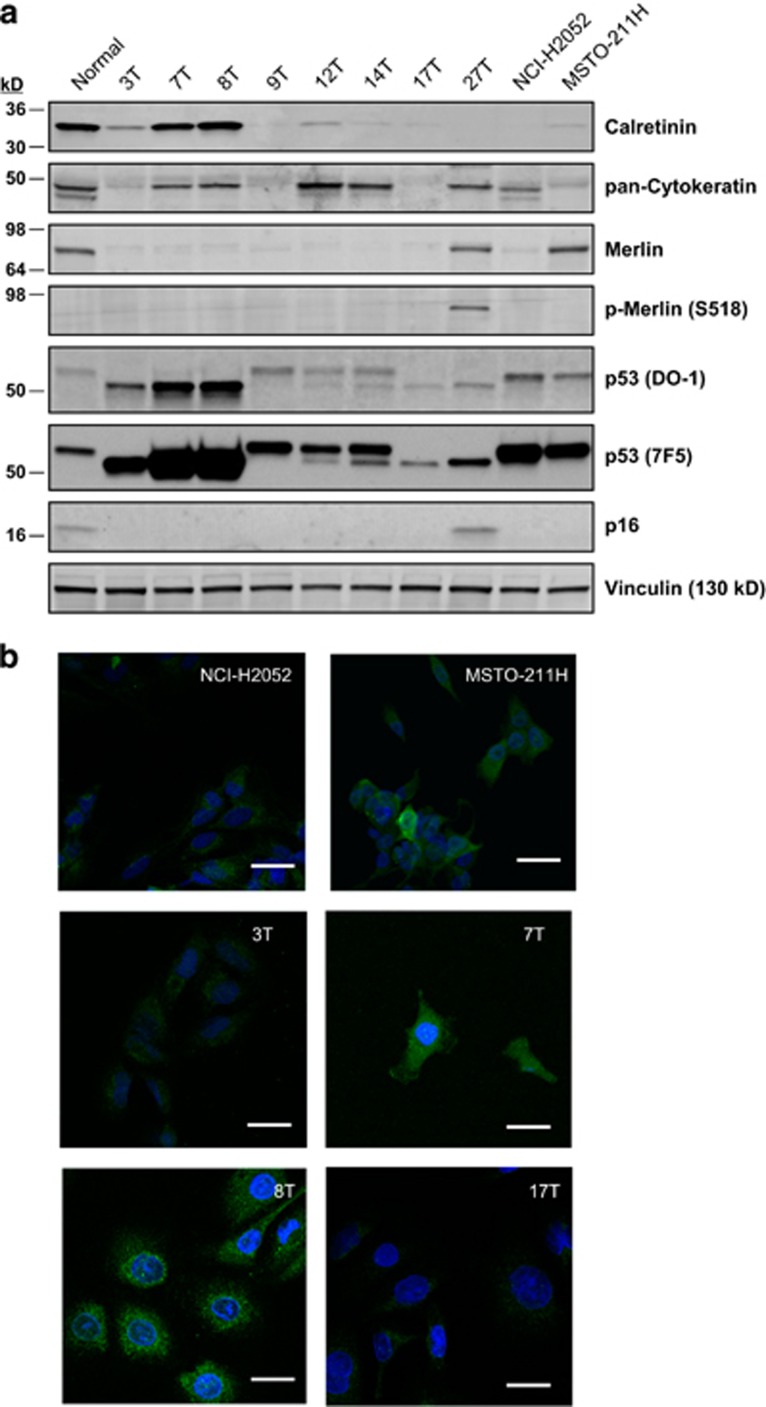
(**a**) Western blot analysis showing different expression of markers and proteins implicated in MM pathogenesis in primary and commercial cell lines. Immunoblotting for MM markers Calretinin and pan-Cytokeratin demonstrates inter-patient variability; loss of Merlin expression in seven out of eight primary cell lines and high levels of Merlin expression in commercial line MSTO-211H; phosphorylation of Merlin in the cell line MESO-27T but not in MSTO-211H; differential expression of p53 isoforms in patient-derived and commercial cell lines (detected by two different anti-p53 antibodies); loss of p16 expression in seven out of eight primary cell lines and commercial cell lines. (**b**) Immunofluorescence staining of commercial and primary mesothelioma cell lines for Calretinin (green), nuclei stained with DAPI (blue). Scale bar indicates 20 *μ*m

**Figure 3:**
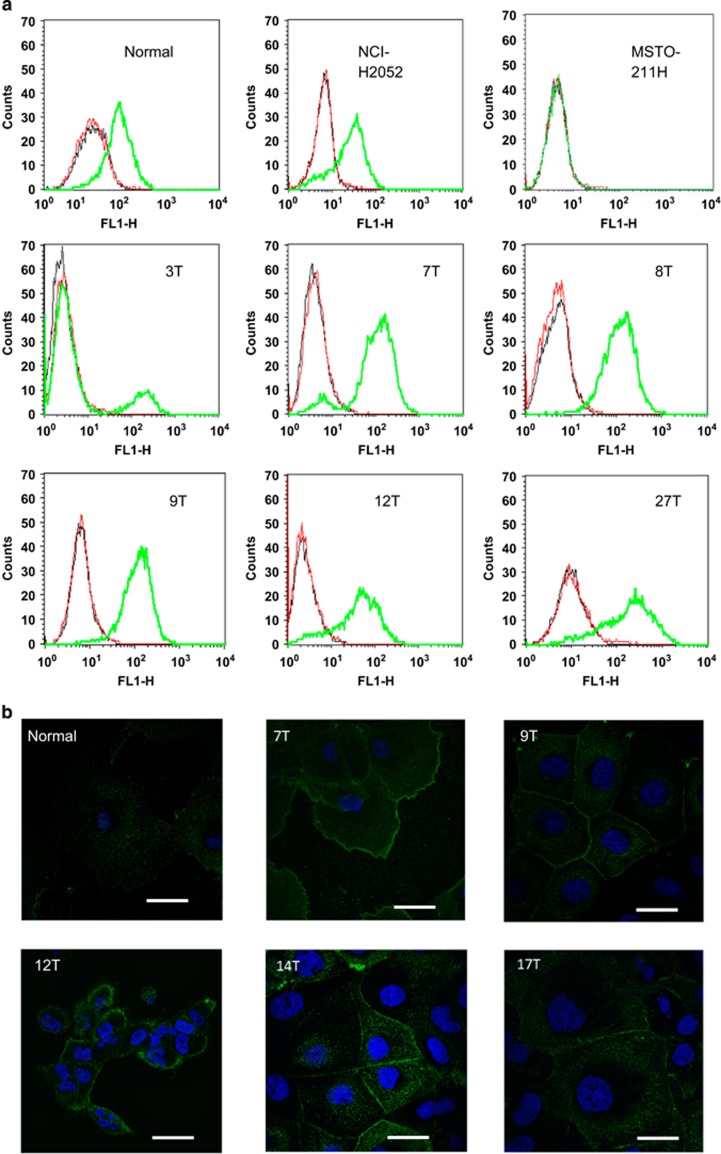
(**a**) Mesothelioma cell lines show differential cell surface expression of Podoplanin. Non-permeabilized normal mesothelial (Normal), commercial lines NCI-H2052 and MSTO-211H or six patient-derived lines (MESO-3T, MESO-7T, MESO-8T, MESO-9T, MESO-12T and MESO-27T) were labeled with either no primary (black), isotype control (red), or mouse anti-Podoplanin antibody (green) and assessed by flow cytometry. Fluorescence intensity is proportional to the amount of Podoplanin on the cell surface. Normal mesothelial cells displayed a low level of Podoplanin expression. Most lines tested showed positive staining with Podoplanin antibody, whereas MESO-3T expressed little and MSTO-211H no Podoplanin on the cell surface. (**b**) Immunofluorescence staining of normal mesothelial and primary mesothelioma cell lines for transmembrane Podoplanin (green), nuclei stained with DAPI (blue). Membrane staining is displayed by primary cell lines. Scale bar indicates 20 *μ*m

A panel of diagnostic markers and proteins associated with MM development was examined in both primary and commercial cell lines. Consistent with immunochemistry results in the clinic (70–80% of mesotheliomas have positive staining for pan-Cytokeratin^18, 19, 20^), western blot analysis showed that five out of eight primary cell lines were strongly positive for pan-Cytokeratin (Figure 2a). The commercial cell line NCI-H2052 expressed pan-Cytokeratin, whereas MSTO-211H cells were negative (Figure 2a). Among the diagnostic markers of MM, Calretinin and Podoplanin have the maximal sensitivity and selectivity.^18, 19, 20^ Calretinin was detected by western blot analysis in normal mesothelial cells, with high levels evident in MESO-7T and MESO-8T. The level of expression in the remaining primary cell lines was significantly lower and appeared similar to that detected in the commercial lines (Figure 2a). Confocal microscopy of immunofluorescently stained cells showed positive staining for Calretinin in all patient-derived cell lines, with the highest level detected in MESO-7T and MESO-8T (Figure 2b). Sequencing of the 46 Cancer Gene Panel (including the tumor suppressor gene *TP53* and proto-oncogenes listed in Supplementary Table 2) in the primary lines, MESO-3T, MESO-7T, MESO-8T, MESO-12T and both commercial cell lines, did not reveal any genetic alterations besides common polymorphisms (data not shown). However, western blot analysis of p53 expression showed that the isoform expressed by normal mesothelial cells was also found in both commercial lines, whereas MESO-9T, MESO-12T, MESO-14T expressed a higher molecular weight and MESO-3T, MESO-7T, MESO-8T, MESO-17T and MESO-27T a lower molecular weight isoform of p53, respectively (Figure 2a). Consistent with the reported loss of the *NF2*-encoded tumor suppressor Merlin in MM clinical specimens,^16, 21, 22^ all primary cell lines except for MESO-27T were Merlin-negative. In contrast, Merlin was highly expressed in MSTO-211H with only low expression observed in NCI-H2052 (Figure 2a). Examination of the *CDKN2A*-encoded protein p16 detected loss of expression in all primary cell lines except for MESO-27T, as well as in the commercial lines. Podoplanin is a mucin-type transmembrane glycoprotein expressed in normal cells^23, 24^ and highly expressed in cancer^25, 26^ with an important role in tumor progression.^24, 25, 26^ Podoplanin was assessed by FACS (Figure 3a) and confocal analysis (Figure 3b) of cells stained with Podoplanin antibody without prior permeabilisation. Primary cell lines showed positive staining, although MESO-3T expressed low levels of Podoplanin on the cell surface. In contrast, MSTO-211H cells were Podoplanin-negative and NCI-H2052 cells displayed expression levels similar to normal mesothelial cells (Figure 3a). Consistent with FACS analysis, confocal microscopy showed positive membrane staining for Podoplanin in freshly derived MM cell lines (Figure 3b).

### Status of tumor suppressor genes *p16*^*INK4A*^*/p14*^*ARF*^ and *NF2*

**Figure 4:**
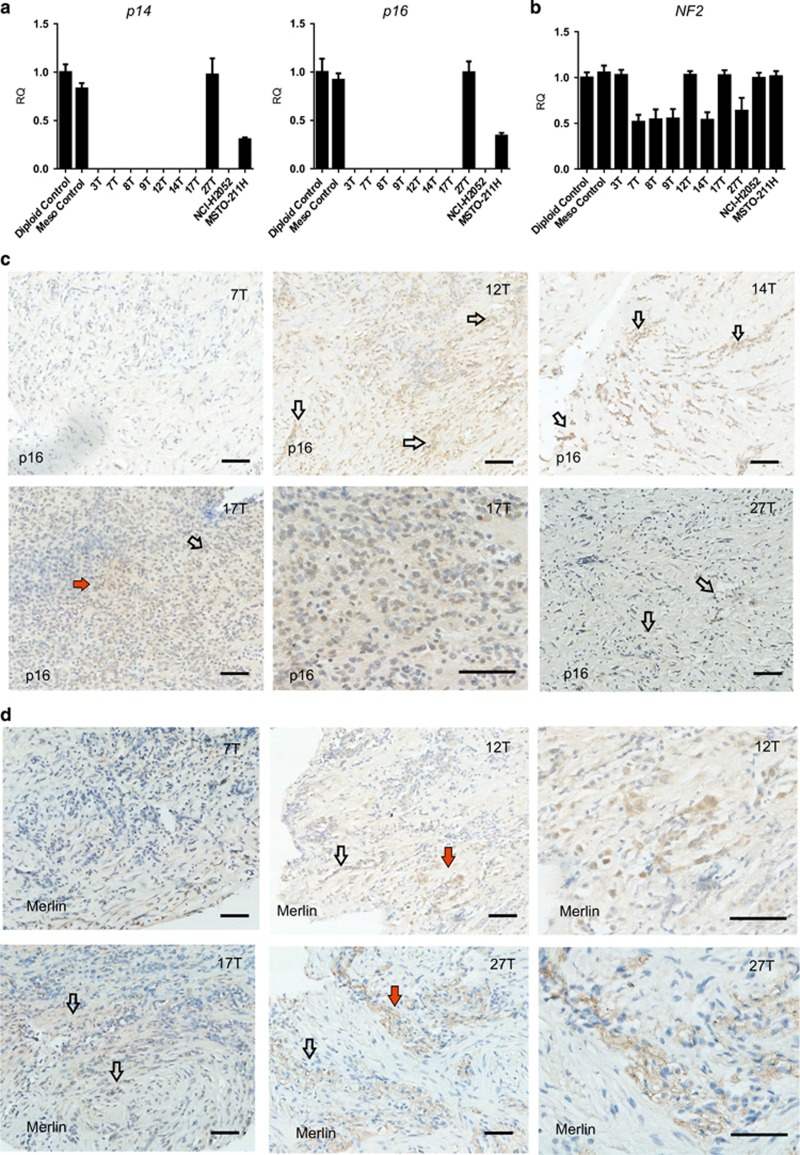
(**a**–**d**) Tumor suppressor genes and proteins in primary and commercial cell lines. (**a**) Relative quantification of *p16*^*INK4A*^*/p14*^*ARF*^ and *NF2* copy number by qPCR in mesothelioma cell lines showing homozygous deletion of *p16*^*INK4A*^*/p14*^*ARF*^ in seven out of eight patient-derived cell lines and in NCI-H2052, but not in MSTO-211H. The graphs show relative quantification of each locus (mean of 2^−ΔΔCT^). (**b**) Relative quantification of *NF2* copy number by qPCR showing heterozygous deletion of *NF2* in MESO-7T, MESO-8T, MESO-9T, MESO-14T and MESO-27T, but not in MESO-3T, MESO-12T, MESO-17T and in the commercial lines NCI-H2052 and MSTO-211H. The graph shows relative quantification of *NF2* copy number (mean of 2^−ΔΔCT^). (**c**) FFPE sections of the patient tumors stained for p16; positively stained areas are marked by arrows, the areas marked by red arrows are shown at higher magnification. Scale bar indicate 20 *μ*m. (**d**) FFPE sections of the patient tumors stained for *NF2* product Merlin; positively stained areas are marked by arrows, the areas marked by red arrows are shown at higher magnification. Scale bar indicates 20 *μ*m

Deleterious alterations of *NF2* and *CDKN2A* (*p16*^*INK4A*^*/p14*^*ARF*^) have been implicated in mesothelioma development^10, 16, 27^ and are of great importance in determining appropriate therapeutic strategies.^16^ We examined the status of *p16*^*INK4A*^*/p14*^*ARF*^ genetic loci in both primary and commercial mesothelioma cell lines (Figure 4a and b). Relative quantification of gene copy number confirmed homozygous deletion of *p16*^*INK4A*^ and *p14*^*ARF*^ in seven out of eight patient-derived cell lines (with the exception of MESO-27T). The commercial cell line MSTO-211H retained the *CDKN2A* gene; however, the copy number was reduced by ~60%, corresponding to the loss of one allele (Figure 4a; http://cancer.sanger.ac.uk/cosmic). Despite the presence of one *p16*^*INK4A*^ allele, p16 protein was not expressed in this cell line (Figure 2a) implying a mechanism of gene silencing.^28, 29^ Neither deletion nor silencing of p16 was found in the MESO-27T cell line as evidenced by both gene copy number and protein expression (Figures 2a and 4a). To explore whether cell lines with p16 loss were derived from p16-negative tumors, matching patient FFPE tumor tissue was stained for p16 (Figure 4c). The results were consistent with known inter-patient variability: tumor 7T was p16-negative, whereas tumors 12T, 14T, 17T and 27T had both p16-positive and -negative tumor cells. Patchy staining for p16 protein may be due to deletion of *p16*^*INK4A*^ in some tumor cells and/or transcriptional silencing of the *p16*^*INK4A*^ locus.

*NF2* copy number was reduced by ~50% in the primary cell lines MESO-7T, MESO-8T, MESO-9T, MESO-12T and MESO-14T compared with normal mesothelial cells, suggesting heterozygous loss of *NF2*. No change in *NF2* copy number was evident in MESO-3T, MESO-12T, MESO-17T or in either commercial cell line (Figure 4b). Similar to p16, the pattern of Merlin expression in patient FFPE tumor sections consisted of both positive and negative areas (Figure 4d). However, the majority of primary cells derived from these tumors were Merlin-negative (Figure 2a); this included cell lines that still retained one or both alleles of *NF2*, suggesting transcriptional silencing^30^ or proteasomal-mediated protein degradation.^31^ MESO-27T was the exception, with expression of both gene and protein (Figures 2a and 4a). However, further examination of MESO-27T showed that Merlin was phosphorylated at S518 (Figure 2a) and is therefore functionally inactive.^32, 33^ In contrast, the commercial line MSTO-211H retained both alleles of *NF2* and expressed non-phosphorylated Merlin protein.

### Primary and commercial cell lines display a different transcriptional profile

**Figure 5:**
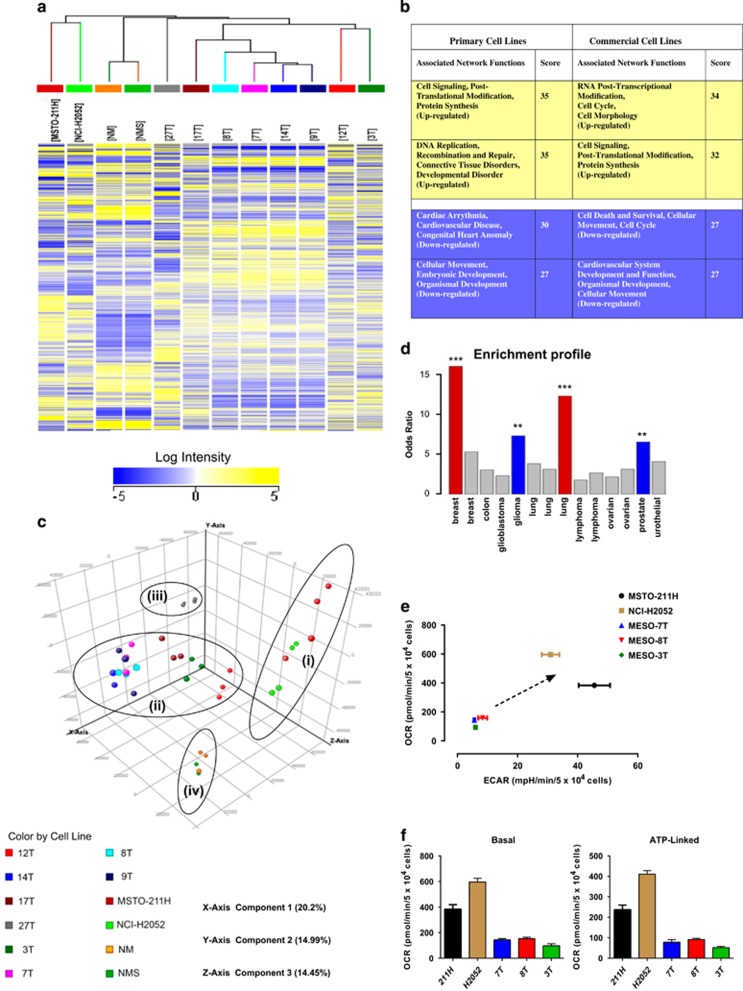
(**a**) Gene expression pattern and hierarchical clustering of control (NM - cells from a single donor and NMS - pool from four donors), commercial (MSTO-211H, NCI-H2052) and primary mesothelioma cell lines (3T, 7T, 8T, 9T, 12T, 14T, 17T, 27T). RNA was extracted from at least three independent cultures for each cell line. The heat-map displays the expression level of the most variable transcripts across the samples (fold change >2 compared with NM control). The legend bar shows the color code for the normalized log intensity values. Two-dimensional hierarchical clustering was performed using the average linkage-clustering method. Relationships among the cell lines are represented by a binary tree (dendrogram). The vertical position of the split gives the distance (dissimilarity) between the tested cell lines. (**b**) Top networks in the transcriptome. Significantly highly represented networks were identified. Networks that were significantly highly represented (*P*≤10^−10^; Fischer's exact test) were identified from the gene list, with significant difference (ANOVA) compared with normal mesothelial cells, using Ingenuity Pathway Analysis software (Ingenuity Systems, Redwood City, CA, USA). The network score describes the probability (*P*=10^-network score^) that the molecules in the network are associated with the data set by chance alone. Networks with a score of +30 were viewed as highly significant (10=minimum score). (**c**) 3D plot of the scores from Principal Component Analysis. Four separate groups corresponding to the hierarchical clustering results were highlighted by circles (i-commercial cell lines, ii- seven primary cell lines, iii- MESO-27T cell line and iv-normal primary mesothelial cells) and present a strong correlation of signals (**d**), Enrichment profile across 15 data sets (the best hit for each data set is plotted) showing high similarity (***) of MM primary cell lines to lung and breast cancer (odds ratio>10) and similarity (**) to glioma and prostate cancer (odds ratio>5) in terms of aberrantly expressed genes. (**e**) Metabolic upregulation of commercial compared with primary cell lines. To assess the difference in metabolism between primary and commercial mesothelioma lines, 5 × 10^4^ cells were seeded in XF24 microplates 24 h prior to real time measurements of oxidative phosphorylation (OCR) and extracellular acidification rate (ECAR) as described in Materials and Methods. Basal values of OCR and ECAR were calculated from mean values generated from three independent experiments normalized to 5 × 10^4^ cells. Data points show mean±S.E.M., *n*=3. Increased metabolism is depicted as a shift highlighted by the arrow. (**f**) Basal and ATP-linked OCR mean values were calculated from three independent experiments normalized to 5 × 10^4^ cells. Bars show mean±S.E.M., *n*=3

Whole-genome transcriptional array analysis showed a common pattern of gene expression in all primary mesothelioma cell lines. Genes statistically significantly up- or downregulated by more than twofold compared with normal cells were used for further analysis. Two-dimensional hierarchical clustering analysis was applied to examine the relationship among the samples in a two-dimensional plot, which clustered samples with similarity. Similarities/differences between cell lines are depicted by the dendrogram (Figure 5a). Strikingly, the primary cell lines clustered together, but were distinct from the commercial cell lines (Figure 5a). In addition, Ingenuity Pathway Analysis revealed that the top networks of significantly highly represented (up- or downregulated) pathways differed in primary and commercial cell lines (Figure 5b). Gene Ontology (GO) term Enrichment Analysis also showed transcriptome differences between primary and commercial cell lines (http://chemoprofiling.org/cgi-bin/bioprofiling/view_details.ProfCom.multi.pl?tmpdir=bioprofiling_7168_1444663897&tool=GOdynamics&org=9606). For example, type I interferon signaling pathway-related genes were enriched in most of the primary but not in the commercial cell lines (Supplementary Figure S2). Principal Component Analysis (PCA) further revealed the relationship between cell lines based on their gene expression pattern (Figure 5c). On the 3D PCA plot, four separate groups displaying a strong correlation of signals are highlighted, which correspond to the hierarchical clustering results. Importantly, MESO-27T, which was the only MM cell line that retained expression of both p16 and Merlin, did not cluster together with the other primary lines.

### The transcriptome of primary MM cell lines is similar to the transcriptional profile of other cancers

There are no large-scale clinically annotated gene expression data sets available for MM. Therefore, to gain new insight into the clinical relevance of the transcriptional profile of primary MM cell lines, we compared their transcriptome with 14 large-scale data sets of gene expression and clinical data for other cancers, including breast, lung, colon, prostate, ovarian cancer, glioma, glioblastoma and lymphomas (http://www.chemoprofiling.org/cgi-bin/GEO/cancertarget/web_run_CT.V1.pl). For each data set, the top genes associated with survival were selected (e.g., top 100 genes, top 300 genes, top 500 genes, based on *P*-value of association). The gene groups were then used as reference knowledge, similar to standard GO data, to perform Enrichment Analysis. Enrichment Analysis demonstrated that the list of genes aberrantly expressed in MM primary cell lines was enriched by genes significantly associated with survival in breast and lung cancer. For example, out of the top 100 genes associated with survival in the METABRIC data set,^34^ 32 were present in the list of aberrantly expressed genes in MM primary cell lines (Supplementary Table 3), which is markedly higher than the expected number (two genes). Consistent with this, the odds ratio of enrichment was 15.99 (*P*<<0.001). The similarity of the aberrantly expressed genes in primary MM cell lines with the gene sets associated with survival in other cancers is summarized in Supplementary Table 4 and Figure 5d.

### Commercial cell lines are metabolically upregulated compared with primary MM cell lines

It is well documented that tumor cells exhibit deregulated metabolism,^35^ which in part explains their ability to adapt to changing conditions within the tumor microenvironment.^36^ To compare the metabolic profile of primary and commercial cell lines, cells were analyzed using a Seahorse Flux Analyser for assessment of both oxidative phosphorylation and glycolysis (Figure 5e and f). Both commercial cell lines exhibited much higher rates of glycolysis compared with primary cells (four- to five-fold), as demonstrated by increased levels of lactic acid release (extracellular acidification). In addition, rates of oxygen consumption (OCR), representative of mitochondrial oxidative phosphorylation (ox-phos), were greatly increased (two- to four-fold) in commercial lines compared with primary cells. These data demonstrate that commercial mesothelioma cells lines have undergone metabolic upregulation to depend heavily on both glycolysis and ox-phos.^37, 38^ Increased metabolism is depicted as a shift highlighted by the arrow (Figure 5e), and increased basal and ATP-linked OCR (Figure 5f). In addition, the metabolic profile of primary cell lines was compared at low and high passage and no difference was detected (Supplementary Figure S3). Modified cellular metabolism clearly challenges the suitability of these long-established MM commercial lines for studies pertaining to metabolic flux, stage of cellular transformation or the ability to forecast metastatic potential.^39^

### Mesothelioma cell lines originate from tumors displaying genomic instability

Although established primary cell lines were authenticated by short tandem repeat (STR) DNA profiling, their profiles were also matched to the original patient tumor genomic DNA (gDNA). The results showed some differences between STR profiles of gDNA from a primary cell line and the original tumor (Supplementary Figure S4). MM is a highly heterogeneous tumor with genomic alterations occurring in malignant cells but usually not in the stroma. In cell lines MESO-3T, MESO-7T, MESO-8T, MESO-14T and MESO-27T allele numerical values for STR loci were different from the original tumor consisting of a mixed cell population. The MESO-7T and MESO-27T cell lines were derived from particularly genetically unstable tumors with four and three loci, respectively, changing the STR size when compared with parental tumor gDNA (Supplementary Figure S4). These differences reflect the high degree of genomic instability of MM with changes also evident while passaging cells in culture. Further alteration of allele numerical values at high passages were noted in MESO-3T, MESO-7T and MESO-14T (Supplementary Figure S4).

Cytogenetic analysis of primary MM cell lines identified both chromosomal structural abnormalities and changes in aneuploidy status, with structural abnormalities commonly seen in chromosomes 1, 2, 3, 6 and 7 (Table 2). Whereas similar abnormalities were observed at early and late passages, in some cell lines further structural abnormalities and a change in aneuploidy status were evident at high passage (MESO-7T, MESO-8T and MESO-27T).

## Discussion

*In vitro* cellular models have significantly contributed to our understanding of tumor biology and response to therapeutic agents.^6, 8, 9, 11, 12^ However, successful translation of promising pre-clinical data to the clinical setting is limited; for example, out of a number of novel therapies recently examined in clinical trials,^40^ to date none has been approved for treatment of mesothelioma.^41^ The development of new therapeutic strategies requires improved pre-clinical models that are more physiologically relevant than long-established, widely used commercial cell lines. Although 2D cell culture models lack the architectural and cellular complexity of real tumors, primary cells, nevertheless, are representative of the patient tumor close to its native state and provide a relatively simple model defined by the ‘robustness' of the system for evaluating new agents in early pre-clinical studies. Generation of primary human mesothelioma cell lines is therefore an important tool for studying the response of this highly chemoresistant tumor to new therapies. Eight new mesothelioma cell lines were established *in vitro* and characterized using complementary multidisciplinary approaches. Clinically relevant markers of mesothelioma, transcriptional and metabolic profiles, as well as status of the tumor suppressor genes *CDKN2A* and *NF2*, the ‘gatekeeper' in MM development,^32^ were examined in the primary cell lines and compared with those of two widely used commercial cell lines. FACS analysis and confocal microscopy of non-permeabilised cells immunostained for Podoplanin^18, 19^ demonstrated that all primary cell lines displayed cell surface expression. In contrast, the commercial cell lines tested Podoplanin-negative or were similar to normal primary mesothelial cells. Another important feature of the primary cell lines was the loss of *CDKN2A* in seven out of eight lines. The *CDKN2A* genetic locus encodes the proteins p14 and p16; p14 activates p53 by rescuing it from proteasome-mediated proteolysis, whereas p16 antagonizes the cyclin-dependent kinases 4 and 6, consequently blocking cell cycle progression.^42, 43^ Although the *TP53* gene is rarely inactivated in MM,^13, 16^ our observation of multiple p53 isoforms with variable expression levels in primary cell lines emphasizes an inter-patient variability characteristic of MM. A predominance of primary cell lines negative for p16 protein suggests that this may have arisen from clonal selection with p16-negative cells growing more readily *ex vivo*. The presence of *p16*^*INK4A*^ and *NF2* genes and their encoded proteins p16 and Merlin in MESO-27T indicates, however, that loss of these proteins is not a prerequisite for successful culturing of MM tumor cells *in vitro*. Alternatively, there is also the possibility that *CDKN2A*/p16 was lost as cells adapted to the culture environment. Either way, the status of key tumor suppressor genes^13, 14, 15^ in cellular models should be taken into consideration in experiments designed to target these pathways.^44^ Interestingly, although the commercial cell line MSTO-211H displayed only heterozygous loss of the *p16*^*INK4A*^ and *p14*^*ARF*^ loci, p16 protein was undetectable in these cells. This suggests an additional mechanism, transcriptional silencing, and exemplifies the possibility of two independent mechanisms simultaneously inactivating key tumor suppressor genes in MM. The *NF2* gene is mutated or homozygously deleted in 40–50% of MM,^16, 32, 45^ although established primary and commercial cell lines show more frequent rates of mutation or undetectable protein.^46^ Comparison of *NF2* gene copy number and its product Merlin, highlighted the importance of transcriptional silencing and posttranslational modification in MM,^46^; in seven out of eight primary cell lines it was observed that, despite at least one allele being retained, Merlin was undetectable. Furthermore, although Merlin was expressed in cell line MESO-27T, the protein was inactivated by phosphorylation.^33^

A striking difference in the transcriptional profile between primary and commercial cell lines was reflected by hierarchical clustering analysis, displaying that commercial cell lines clustered together and completely separately from primary cell lines. MESO-27T was positioned close to the other primary cell lines, but was not included in the same cluster; this is perhaps not surprising, considering that MESO-27T, unlike the other primary cell lines, expresses p16 and Merlin. Another very important difference between the primary and commercial cell lines was unveiled by examining their metabolic profiles. Commercial cell lines exhibited increased metabolism compared with both low- and high-passage primary MM cells, with a shift towards a more glycolytic phenotype. The commercial cell lines would therefore be an inappropriate model for studies exploring the cellular response to metabolic perturbation agents, including, for example, strategies based on synthetic lethality.^47^ Metabolic profiling of cultured tumor cells is therefore essential for validation of cell-based *in vitro* models.

The widely reported genomic instability of MM should also be considered when cellular models are being developed. In the cell line MESO-7T, TH01 (a more ‘stable' locus^48^) and three other loci had altered STR values, with a further two loci altered at high passage, implying genomic instability of these cells. Although we cannot fully rule out that these changes existed before cell isolation, no peak imbalance (indicative of a mixed cell population in the parental tumor sample) was observed on the electropherogram (Supplementary Figure S5), raising the possibility that these additional genetic changes occurred while the primary cell line MESO-7T was in culture. Consistent with this, additional chromosomal structural abnormalities and a decrease in aneuploidy were observed in MESO-7T at high passage. These findings highlight that monitoring of STR profiles and aneuploidy status is essential to determine whether further genetic alterations are acquired during long-term culture.

GO term Enrichment Analysis revealed a similarity between primary MM cell lines and other cancers. In particular, genes highly associated with survival in breast and lung cancer were enriched in the transcriptome of primary MM cell lines. Significantly, this approach has proved to be a powerful tool to uncover the potential clinical relevance of transcriptome profiles in MM, and could also be of benefit in other tumor types where no clinically annotated gene expression data sets are available.

The key differences between primary and commercial MM cell lines presented here highlight the importance of careful evaluation of a pre-clinical cellular model in terms of its suitability for a particular type of research. Although the generation of primary cell lines is both challenging and labor intensive, this strategy yields *in vitro* models that are superior for translation of pre-clinical data to the clinical setting. Our observation of a significantly different transcriptome profile displayed by commercial lines and their marked metabolic shift toward a greater glycolytic phenotype highlights the limited suitability of long-established cell lines for translational research. Furthermore, marked inter- and intra-patient MM heterogeneity in terms of tumor suppressor gene status was also evident in freshly derived cell lines, highlighting the need for careful pre-evaluation of the suitability of MM cell-based models used in pre-clinical testing of agents targeting p16, p53, Merlin and related pathways.

## Materials and Methods

### Collection of clinical specimens

All patients underwent surgery for radical decortication without prior chemotherapy or radiotherapy. The resected mesothelioma specimens were collected with patient consent. Solid tumors were immediately immersed in ice-cold RPMI-1640 supplemented with penicillin (100 U/ml), streptomycin (100 *μ*g/ml) and 10% FBS. Samples were transported to the laboratory for primary cell culturing within 1 h of collection. Human investigations were performed after Research Ethics Committee approval (LREC 08/H0406/226).

### Establishment of primary cell lines

Tumor tissue was mechanically dissociated under sterile conditions and transferred into serum-rich dissection media (RPMI-1640 media supplemented with 10% FBS); cell release was encouraged by mixing of the tissue suspension. Cells were collected by centrifugation at 300 × *g* followed by red blood cell lysis. Cells were seeded in gelatin-coated flasks at 5 × 10^4^/cm^2^ and maintained in RPMI-1640 growth media supplemented with l-glutamine (2 mM), penicillin (100 U/ml), streptomycin (100 *μ*g/ml), hEGF (20 ng/ml), hydrocortisone (1 *μ*g/ml), heparin (2 *μ*g/ml) and 2% FBS at 37 °C and 5% CO_2_. Differential trypsinisation with 0.05 and 0.1% trypsin was used to remove fibroblasts and select mesothelial cells, respectively. Culture purity varied from 97% (owing to occasional non-proliferating fibroblasts) to 100% of malignant mesothelial cells. After the cell lines were established they were cultured in RPMI-1640 growth media supplemented with l-glutamine (2 mM), penicillin (100 U/ml), streptomycin (100 *μ*g/ml), hEGF (20 ng/ml), hydrocortisone (1 *μ*g/ml), heparin (2 *μ*g/ml) and 10% FBS at 37 °C and 5% CO_2_. Cells were monitored regularly for their morphology. All cell lines were authenticated by STR DNA profiling (LGC Standards, Teddington, UK) and matched to the original tumor tissue. All cell culture reagents were from Life Technologies (Paisley, UK), except for hydrocortisone and heparin (Sigma-Aldrich, Dorset, UK).

### Commercial cell lines

Two mesothelioma cell lines, MSTO-211H and NCI-H2052, were obtained from the American Type Culture Collection (Manassas, VA, USA) and cultured in RPMI-1640 supplemented with l-glutamine (2 mM), penicillin (100 U/ml), streptomycin (100 *μ*g/ml) and 10% FBS in a 5% CO_2_ humidified incubator at 37 °C. Adult human primary omental mesothelial cells (single or multi-donor) purchased from Cambridge Bioscience (Cambridge, UK) served as normal controls (Normal) and were maintained in culture in growth medium (Medium 199, 10% FBS, 20 ng/ml hEGF, 100 U/ml penicillin, 100 *μ*g/ml streptomycin) for up to three passages.

### Electron microscopy

For transmission electron microscopy (TEM), cell pellets were fixed in 2% glutaraldehyde in 0.1 M sodium cacodylate buffer (pH 7.4) at 4 °C overnight and postfixed with 1% osmium tetroxide/1% potassium ferrocyanide for 1 h at room temperature. After fixation, cells were stained en bloc with 5% aqueous uranyl acetate overnight at room temperature, dehydrated in a series of alcohols and embedded in Taab epoxy resin (Taab Laboratories Equipment Ltd., Aldermaston, UK). Confluent cultures, in plastic dishes, were also processed *in situ* before the plastic was removed, with propylene oxide, and the blocks re-embedded for orthogonal sectioning. Ultra-thin sections were stained with lead citrate and recorded using a Megaview 3 digital camera and iTEM software (Olympus Soft Imaging Solutions GmbH, Münster, Germany) in a Jeol 100-CXII electron microscope (Jeol UK Ltd., Welwyn Garden City, UK).

For scanning electron microscopy, cells on glass coverslips were processed to absolute alcohol, as for TEM, transferred to hexamethyldisilazane and air-dried before being sputter-coated with a 15 nm layer of gold and examined in a FEI Quanta FEG 250 electron microscope (FEI Europe, Eindhoven, the Netherlands).

### Flow cytometry analysis

MM cell suspension (1 × 10^6^/ml) was incubated at 4 °C for 1 h with anti-Podoplanin antibody (1 : 100, Santa Cruz, Middlesex, UK; sc-59347) or isotype control antibody (Mouse anti-BCL-2). Cells were pelleted, washed and re-suspended in normal medium containing goat anti-mouse IgG conjugated with FITC (1 : 100, Dako, Cambridge, UK) and incubated for 1 h at 4 °C. Cells were washed and re-suspended in PBS before analysis by BD FACS Calibur; data were acquired and analyzed with CellQuest software.

### Western blot analysis

Cells were lysed for 20 min on ice in lysis buffer (50 mM Tris pH 7.5, 0.5 M NaCl, 1% NP40, 1% sodium deoxycholate, 0.1% SDS, 2 mM EDTA, and complete protease inhibitors (Roche, Basel, Switzerland)), briefly sonicated and the resultant lysate clarified by centrifugation at 1000 × *g* for 10 min. Protein content was measured by Bradford assay and 20 *μ*g protein was loaded onto 4–20%, Criterion TGX gels (Bio-Rad Laboratories, Hemel Hempstead, UK). Proteins were blotted onto polyvinylidene difluoride membranes (Millipore, Watford, UK). Immunoblotting was done after blocking with 5% non-fat milk with the following primary antibodies: anti-Merlin mAb D1D8 and anti-p53 (7F5) (Cell Signaling, Hitchin, UK), anti-p53 (DO-1), and anti-pan-Cytokeratin (H-240) (Santa Cruz), anti-Vinculin mouse, anti-Merlin (phospho-S518) rabbit (Abcam, Cambridge, UK), anti-p16 (G175–405) and anti-Calretinin mouse (BD Bioscience, Oxford, UK). Bands were detected using fluorescently-labeled secondary antibodies (LiCor Biosciences, NE, USA) according to the manufacturer's recommendations. The membranes were subsequently scanned with an Odyssey Infrared Imager (LiCor Biosciences) and converted to greyscale.

### Immunohistochemistry and immunofluorescence

Immunohistochemistry of formalin-fixed, paraffin-embedded tissues from human explants was performed with the Histostain-Plus detection System according to the manufacturer's protocol (Life Technologies). Sections were dewaxed and rehydrated, and antigen retrieval was performed by heating the slides in 10 mM citric acid buffer (pH 6.0) for 15 min. Sections were incubated overnight at 4 °C in a humidified chamber with a 1 : 10 dilution of antibody to p16 (BD) and 1 : 50 NF2 (Santa Cruz). Control IHC experiments (data not shown) were performed without primary antibody. All sections were counterstained with Gill's haematoxylin and mounted for digital slide scanning.

For immunofluorescence, isolated cells were first fixed in 4% paraformaldehyde and where indicated permeabilized with 0.1% Triton in PBS-Tween (0.1% vol/vol), prior to blocking in 5% goat serum in PBS-Tween. Cells were then incubated with primary antibody 1 : 50 anti-Podoplanin (Santa Cruz) without prior permeabilization; 1 : 50 anti-Calretinin (Cell Signaling) overnight at 4 °C. Cells were washed three times for 10 min each with 1 × PBS, incubated for 1 h with secondary antibody (in blocking solution), washed three times with 1 × PBS, and counterstained with DAPI and mounted for confocal microscopy.

### RNA microarrays

Total RNA was extracted by TRIzol (Life Technologies). Hybridization to 60 K whole human genome microarray gene expression chips was conducted following manufacturer's protocol (Agilent Technologies, Berkshire, UK). In brief, total RNA samples were Cy3-labeled using Agilent Low Input Quick Amp 1-colour Labelling Kit. The level of dye incorporation was evaluated by spectrophotometry (Nanodrop ND1000, LabTech, Uckfield, UK). Labeled RNA was then fragmented and diluted (v/v) in hybridization buffer. Hybridization to 60 K high-density oligonucleotide microarray slides was performed in a microarray hybridization oven overnight at 65 °C. Following hybridization, the slides were rinsed and immediately scanned using a DNA Microarray Scanner (Model G2505C, Agilent Technologies).

### Analysis of microarray data

The raw data were uploaded into Agilent's GeneSpring Software, normalized and fold changes calculated. For each cell line the probes with an absolute twofold-change (*P*<0.05) in mRNA expression compared to normal mesothelial cells were included in subsequent analyses. These were subjected to Anova unequal variations test with Benjamin–Hochberg corrections. Significant (*P*<0.05) changes were subjected to hierarchical clustering with average linkage. The clustered heat-map was visualized using GeneSpring. Significantly highly represented networks were identified using Ingenuity Pathway Analysis software (Ingenuity Systems, Redwood City, CA, USA). The PCA (statistical procedure elucidating the covariance structure of a set of variables) was performed for the three most changing components to identify the principal directions in which the mRNA changes varied. The data were projected on three axes (components), ordered by decreasing significativity; the first principal component explains 20.2% of the variance, the second explains 14.99% and the third explains 14.45% of the variations in expression. GO term Enrichment Analysis was conducted as described previously.^49, 50^

### Relative quantification of gene copy number by real-time PCR

gDNA was extracted using QIAamp DNA Blood Mini Kit (Qiagen, Manchester, UK) according to the manufacturer's instructions. An assay based on the paralogue ratio test and real-time PCR was used to determine the DNA copy number as described previously.^51^

Real-time PCRs were performed on the ABI 7500 Fast system (Applied Biosystems Foster City, CA, USA). Each 10-*μ*l reaction contained 3.4 *μ*l of gDNA (10 ng), 0.3 *μ*l of each of the two primers required for each loci (20 pM), 0.2 *μ*l of each of the probes required (50–200 nM), and 5 *μ*l of Genotyping MasterMix (Applied Biosystems). The PCRs began with 2 min at 50 °C, 10 min at 95 °C, followed by a cycle consisting of 15 s at 95 °C and 60 s at 60 °C. Fifty cycles were performed. C_T_ values were determined using the automatic threshold settings. gDNAs from diploid control (Roche, human gDNAs, catalog #11691112001) and normal mesothelial cells were used as controls. The ΔC_T_ was calculated for each of the reactions using the following equation: C_T_ gene 1 (gene of common DNA loss)−C_T_ gene 2 (reference)=ΔC_T_. The mean ΔC_T_ from at least three reactions was used for subsequent analysis. The relative quantification of copy number was calculated using the 2^−ΔΔCT^ value. Primers and probes are listed below:

### Cell metabolism assays

Rates of oxidative phosphorylation and glycolysis were determined by measuring the rate of OCR and extracellular acidification rate (ECAR), respectively, using a Seahorse XF24 extracellular flux (XF) analyser (Seahorse Bioscience, Billerica, MA, USA).

Commercially available and primary MM cell lines were seeded in a XF24 cell culture microplate at 5 × 10^4^ cells/well 24 h before the assay and incubated at 37 °C under 5% CO_2_ in a humidified atmosphere. Primary lines were seeded on gelatin coated microplates. 24 h prior to assay, 1 ml of Seahorse calibrant was added to each well of a Seahorse XF24 utility plate and the probes on the sensor cartridge were allowed to soak at 37 °C under CO_2_-free conditions. 1 h prior to assay, cells were washed 3 × in unbuffered bicarbonate and serum-free DMEM (pH 7.4) containing 1 mM sodium pyruvate, 11 mM glucose and 2 mM glutamax; then covered in 675 *μ*l of the same media, and incubated for 1 h at 37 °C under CO_2_-free conditions. The cell microplate was then loaded into the Seahorse XF24 and assayed to measure basal OCR and ECAR.

### Cytogenetic analysis

Chromosome preparations of the cells were made as follows: Colcemid (Gibco Invitrogen, Paisley, UK; 10 *μ*g/ml in PBS) (0.3 *μ*g/ml final concentration) was added to semi-confluent actively dividing cultures 4 h before harvesting. Metaphase cells were harvested by trypsinizing, treated with a hypotonic solution (deionised water) for 15 min at 37 °C and fixed twice in acetic acid (one part)/methanol (three parts). Cells were dropped onto clean slides, heat-aged, treated with trypsin and stained with Leishman stain to produce G-banded chromosomes. In total, 4–10 metaphase spreads were analyzed from each culture, depending on availability, for numerical and structural rearrangements.

## Figures and Tables

**Table 1:** History of asbestos exposure, histopathology results and clinical diagnosis of MM patients included in the study

**Patient sample/cell line ID**	**Age**	**Sex**	**Asbestos exposure**	**Histopathology**	**Clinical diagnosis**
MESO-3T	56	Male	Kitchen fitter and carpenter, exposed to asbestos through employment, asbestos fiber identified in background lung tissue after decortication.	Epithelioid malignant mesothelioma	Left radical pleural decortication. Epithelioid malignant mesothelioma, stage at least pT2, pN0, pMx.
MESO-7T	70	Male	Building contractor, exposed to asbestos through employment, asbestos fiber identified in lung parenchyma after decortication.	Biphasic malignant mesothelioma	Left radical pleural decortication with multiple station nodes. Biphasic malignant mesothelioma, stage pT3, pN2, pMx, R1.
MESO-8T	77	Male	No known asbestos exposure and no asbestos bodies found in lung parenchyma after decortication.	Tumor is predominantly epithelioid with sarcomatoid foci	Left radical pleural decortication. Biphasic malignant mesothelioma, stage pT4, pN2, R1.
MESO-9T	62	Female	Teacher, no asbestos exposure, no asbestos bodies identified in lung tissue post-decortication.	Epithelioid malignant mesothelioma	Left radical pleural decortication. Epitheliod malignant mesothelioma, stage pT4, pN2, R1.
MESO-12T	78	Male	Retired engineer, no known asbestos exposure, no asbestos bodies in specimen post-decortication.	Epithelioid malignant mesothelioma	Right radical pleurectomy and decortication. Epithelioid malignant mesothelioma, stage pT4, pN2, pMx, R1.
MESO-14T	59	Male	Building trade, was cutting asbestos sheets when working, strong exposure.	Epithelioid malignant mesothelioma	Left pleurectomy and decortication. Epithelioid malignant mesothelioma, stage pT2, pN2, pMx, R1.
MESO-17T	72	Male	Carpenter, has been exposed to asbestos, moderate exposure.	Epithelioid malignant mesothelioma	Right radical pleurectomy/ decortication. Epithelioid malignant mesothelioma, stage pT2 (at least), pN2, pMx, R2.
MESO-27T	65	Male	Retired tin box factory worker, has been exposed to asbestos all his working life, strong exposure.	Epithelioid malignant mesothelioma	Right radical decortication of pleura. Epithelioid malignant mesothelioma, stage pT3, pN2, R1.

**Table 2:** Cytogenetic analysis of primary MM cell lines

**Cell line**	**Passage**	**Chromosome count**	**Structural abnormalities of chromosomes**	**Overview**
MESO-3T	Low	~66–>200	Structural abnormalities present.	Range of ploidy levels seen at both low and high passage
	High	~59–>200	Structural abnormalities present.	but no overall change between low and high. Structural abnormalities are present (particularly chromosome 1).
MESO-7T	Low	69–78	1, 3, 6, 7, 8, 9, 10, 11, 13, 17	Decrease in aneuploidy between low and high passage.
	High	43	1, 2, 4, 5, 11, 15	Different structural abnormalities seen at high passage only.
MESO-8T	Low	63–67	1, 3, 6, 7, 8, 9, 13, 21	Increase in aneuploidy between low and high passage;
	High	73–79	1, 3, 6, 7, 8, 9, 10, 13. Doubling of some structural abnormalities.	doubling of some structural abnormalities seen at low passage. Some abnormalities seen only at low passage and vice-versa.
MESO-12T	Low	46	1, 2, 6, 9, 11, 12, 14, 17, 20, 22	No change in aneuploidy. Some minor changes to structural
	High	46	1, 2, 6, 7, 8, 9, 11, 12, 14, 16, 17, 22	rearrangement between low and high passage.
MESO-14T	Low	47–48	1, 2, 6, 7, 8, 9, 11, 12, 16, 17	No significant change in aneuploidy. Some minor changes to
	High	46–47	1, 2, 6, 7, 8, 9, 11, 12, 17	structural rearrangement between low and high passage.
MESO-27T	Low	72–74	Y, 1, 2, 3, 7, 9, 11, 12, 13, 14, 16, 17, 19, 20	General decrease in aneuploidy at high passage. Some minor changes to structural rearrangement between low and high
	High	65–73	Y, 1, 3, 7, 8, 13, 14, 16, 17, 19, 22	passage.
MESO-9T	Low	~46	Structural abnormalities present.	Evidence of doubling of chromosome number; may be due to cultural tetraploidy.
MESO-17T	Low	44–46	8, 19. Missing a Y-chromosome and one chromosome 6.	Less complexity with only a single structural abnormality in each cell along with the loss of whole chromosomes. Evidence of a doubling of chromosome number; may be due to cultural tetraploidy.

Where indicated, actively growing MM cells were analyzed at low and high passage.

**Table:** 

**Target locus**	**Location**	**Primer probe**	**Sequence**
p16	Chromosome 9: 21967752..21994491, complement	Forward primer Reverse primer Probe	5′-AACATGGTGCGCAGGTTCTT-3′ 5′-TGAACCACGAAAACCCTCACT-3′ 5′-6-FAM-CCCTCCGGATTCG-MGB-3′
p14	Chromosome 9: 21967752..21994491, complement	Forward primer Reverse primer Probe	5′-GCGGTCCCTCCAGAGGAT-3′ 5′-CGGTGCTGGCGGAAGA-3′ 5′-6-FAM-TGAGGGACAGGGTCG-MGB-3′
NF2	Chromosome 22: 29999545..30094589	Forward primer Reverse primer Probe	5′- GCCAGGCCCTGCTAGATAGC-3′ 5′-AACCTGTCCCCAAAATTACAAGAC-3′ 5′-6-FAM- CCCCGTGGCATTAC-MGB-3′
AldoB (aldolase B, reference)	Chromosome 9: 101420560..101435780, complement	Forward primer Reverse primer Probe	5′-TTTCCACGAGACCCTCTACCA-3′ 5′-CTTTTCCTTGAGGATGTTTCTGAAC-3′ 5′-6-FAM- AAGGACAGCCAGGGAA-MGB-3′
Svil (supervillin, reference)	Chromosome 10: 29457338..29736935	Forward primer Reverse primer Probe	5′-GCCTGCGGAGCGTCAA-3′ 5′-GGCACGGCGCTGTTGT-3′ 5′-6-FAM-ACGGAACAGAACTCT-MGB-3′

## Abbreviations

MM: malignant mesothelioma
FACS: fluorescence-activated cell sorting
FFPE: formalin-fixed paraffin-embedded
gDNA: genomic DNA
GO: gene ontology
PCA: principal component analysis
STR: short tandem repeat
FBS: fetal bovine serum
hEGF: human epidermal growth factor
TEM: transmission electron microscopy
FITC: fluorescein isothiocyanate
PBS: phosphate-buffered saline
EDTA: ethylenediaminetetraacetic acid
IHC: immunohistochemistry
IF: immunofluorescence
DAPI: 4',6-diamidino-2-phenylindole
PCR: polymerase chain reaction
C_T_: cycle threshold
OCR: oxygen consumption rate
ECAR: extracellular acidification rate
ATP: adenosine triphosphate
