# Supplementary material for: Molecular profiling reveals primary mesothelioma cell lines recapitulate human disease
Source: Cell Death Differ. 2016 Feb 19;23(7):1152–64. doi: 10.1038/cdd.2015.165 (PMC4946883; doi:10.1038/cdd.2015.165)
Supplement: Supplementary Figure S1 [file cdd2015165x1.pdf]

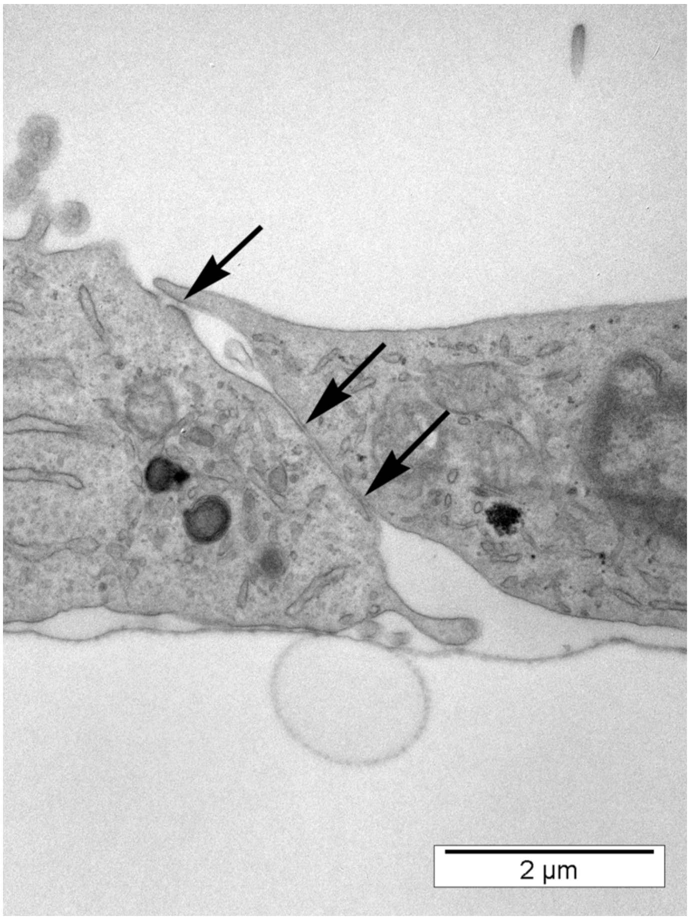

Supplementary Figure S1. Tight intercellular junctions (arrows) in primary mesothelial culture MESO-27T.
