# Supplementary material for: Molecular profiling reveals primary mesothelioma cell lines recapitulate human disease
Source: Cell Death Differ. 2016 Feb 19;23(7):1152–64. doi: 10.1038/cdd.2015.165 (PMC4946883; doi:10.1038/cdd.2015.165)
Supplement: Supplementary Figure S2 [file cdd2015165x2.pdf]

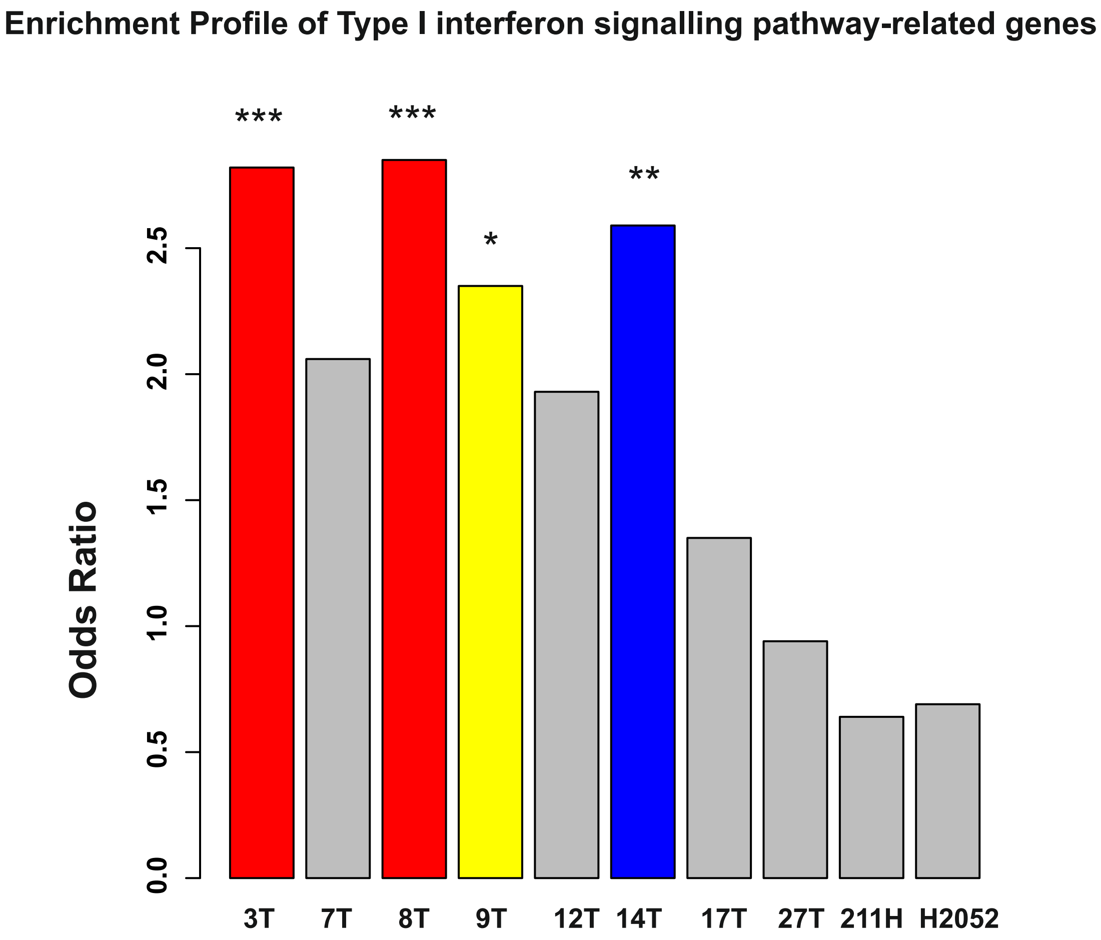

Supplementary Figure S2. GO Term Enrichment profile of the category ‘Type I interferon signalling pathway-related genes’ in primary and commercial cell lines and full statistical details of datasets used as reference. In contrast to primary cell lines, commercial cell lines MSTO-211H and NCI-H2052 display no enrichment for the type I interferon signalling pathway, *** indicates $p<0.001$, ** $p<0.01$, * $p<0.05$.
