# Supplementary material for: Molecular profiling reveals primary mesothelioma cell lines recapitulate human disease
Source: Cell Death Differ. 2016 Feb 19;23(7):1152–64. doi: 10.1038/cdd.2015.165 (PMC4946883; doi:10.1038/cdd.2015.165)
Supplement: Supplementary Figure S3 [file cdd2015165x3.pdf]

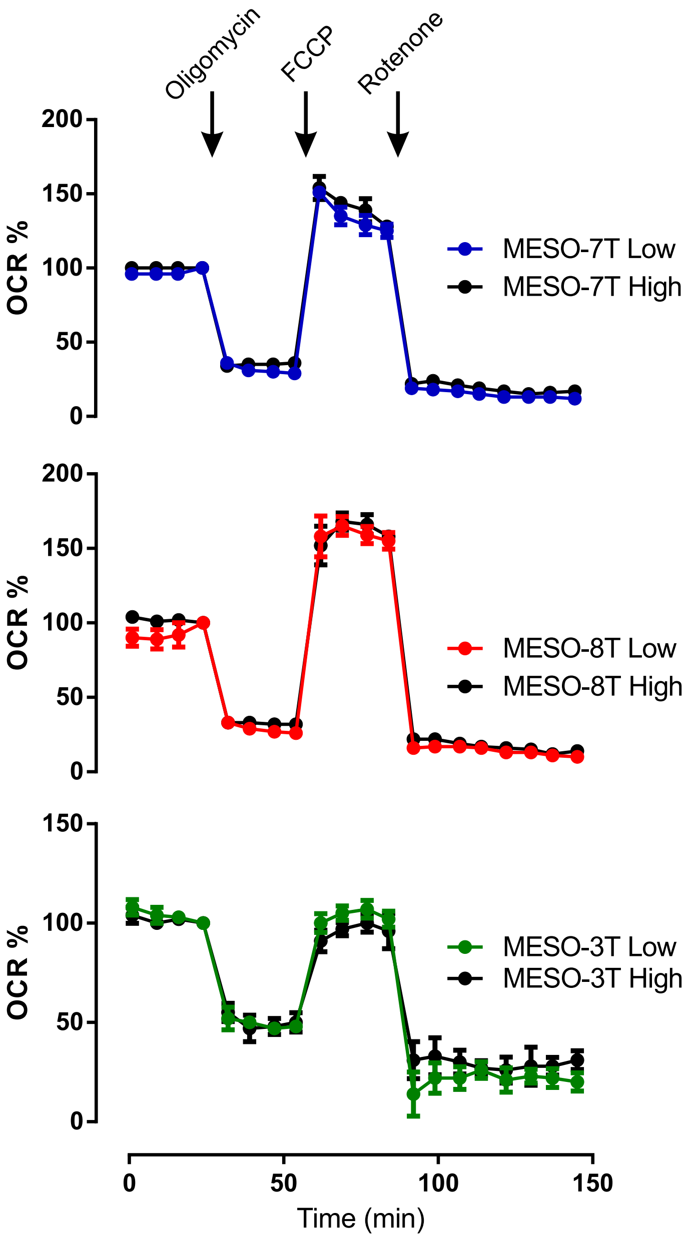

Supplementary Figure S3. Comparison of the metabolic profile of low and high passage primary cell lines. To assess the difference in metabolism between low and high passage primary mesothelioma lines, 5 x 10^4 cells were seeded in XF24 microplates 24 h prior to real time measurements of oxidative phosphorylation (OCR) as described in Materials and Methods. Oligomycin, FCCP and Rotenone were sequentially injected to generate a standard mitochondrial stress test, which generated a trace that was identical between low and high passage MESO-7T, -8T and 3T cells. Data shown is from one experiment representative of 3 independent experiments. Data points show mean ± SEM, n = 5 (technical repeats).
