# Supplementary material for: Molecular profiling reveals primary mesothelioma cell lines recapitulate human disease
Source: Cell Death Differ. 2016 Feb 19;23(7):1152–64. doi: 10.1038/cdd.2015.165 (PMC4946883; doi:10.1038/cdd.2015.165)
Supplement: Supplementary Figure S4 [file cdd2015165x4.pdf]

Powerplex16 Loci	Sample 3T Genomic	Sample MESO-3T LOW	Sample MESO-3T HIGH
AMELO	XY	XY	XY
D3	15,18	18,18	18,18
TH01	6,9.3	6,9.3	6,9.3
D21	31,33.2	31,33.2	31,33.2
D18	13,14	13,14	13,14
PentaE	7,13	7,13	7,13
D5	13,13	13,13	13,13
D13	11,12	11,12	11,11
D7	10,11	10,11	10,11
D16	11,13	11,13	11,13
CSF	10,11	10,11	10,11
PentaD	10,10	10,10	10,10
VWA	17,17	17,17	17,17
D8	11,15	11,15	11,15
TPOX	8,11	8,11	8,11
FGA	23*,24	24,24	24,24

Powerplex16 Loci	Sample 7T Genomic	Sample MESO-7T LOW	Sample MESO-7T HIGH
AMELO	XY	XY	XY
D3	14,18	14,18	14,18
TH01	6,9.3	6,6	6,6
D21	29,30	29,30	29,30
D18	13,20	13,20	13,13
PentaE	14,17	14,17	14,14
D5	11,14	11,11	11,11
D13	8,11	11,11	11,11
D7	10,10	10,10	10,10
D16	11,12	11,12	11,12
CSF	10,12	12,12	12,12
PentaD	11,12	11,12	11,12
VWA	14,18	14,18	14,18
D8	10,13	10,13	10,13
TPOX	8,8	8,8	8,8
FGA	24,25	24,25	24,25

Powerplex16 Loci	Sample 8T Genomic	Sample MESO-8T LOW	Sample MESO-8T HIGH
AMELO	XY	XY	XY
D3	14,18	18,18	18,18
TH01	7,9	7,9	7,9
D21	30,30	30,30	30,30
D18	14,17	14,17	14,17
PentaE	7,10	7,10	7,10
D5	10,12	10,12	10,12
D13	10, 12*	10, 10	10, 12
D7	10,10	10,10	10,10
D16	11,12	11,12	11,12
CSF	10,14	10,14	10,14
PentaD	9,11	9,11	9,11
VWA	16,19	16,19	16,19
D8	13,15	13,15	13,15
TPOX	8,9	8,9	8,9
FGA	21,24	21,24	21,24

Powerplex16 Loci	Sample 14T Genomic	Sample MESO-14T LOW	Sample MESO-14T HIGH
AMELO	XY	XY	XY
D3	14,18	18,18	18,18
TH01	9,9.3	9,9.3	9,9.3
D21	29,30	29,30	30,30
D18	13,18	13,18	13,18
PentaE	7,12	7,12	7,12
D5	11,11	11,11	11,11
D13	11,11	11,11	11,11
D7	10,14	10,14	10,14
D16	9,11	9,11	9,11
CSF	11,12	11,12	11,12
PentaD	9,12	9,12	12,12
VWA	16,18	16,18	16,18
D8	14,15	14,15	14,15
TPOX	8,9	8,9	8,9
FGA	24,24	24,24	24,24

Powerplex16 Loci	Sample 27T Genomic	Sample MESO-27T LOW	Sample MESO-27T HIGH
AMELO	XY	XY	XY
D3	15,17	15,17	15,17
TH01	9.3,9.3	9.3,9.3	9.3,9.3
D21	29,30	29,30	29,30
D18	13,21	13,13	13,13
PentaE	7,10	7,10	7,10
D5	11,13	11,13	11,13
D13	12,13	12,13	12,13
D7	10,11	10,11	10,11
D16	12,13	13,13	13,13
CSF	11,13	11,13	11,13
PentaD	9,9	9,9	9,9
VWA	16,17	16,17	16,17
D8	8,13	8,13	8,13
TPOX	8,8	8,8	8,8
FGA	23,25	23,23	23,23

Supplementary Figure S4. Difference in numerical values for STR loci between the original tumors and cell lines derived from those tumors, and between low (<10) and high (30-50) passage cells in culture (* indicates Peak Area Difference (n) Below-threshold peak stutter).
