# Supplementary material for: Molecular profiling reveals primary mesothelioma cell lines recapitulate human disease
Source: Cell Death Differ. 2016 Feb 19;23(7):1152–64. doi: 10.1038/cdd.2015.165 (PMC4946883; doi:10.1038/cdd.2015.165)
Supplement: Supplementary Figure S5 [file cdd2015165x5.pdf]

Tumor 7T genomic DNA

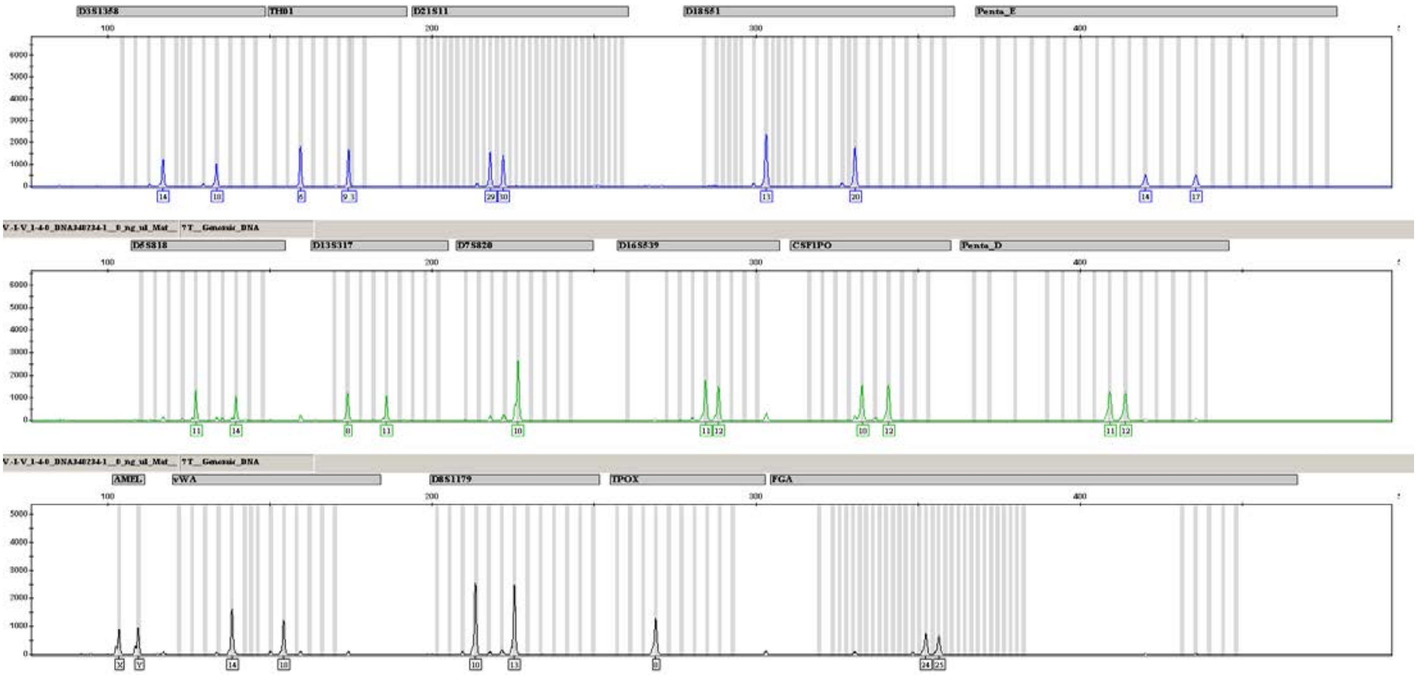

Primary cell line MESO-7T genomic DNA

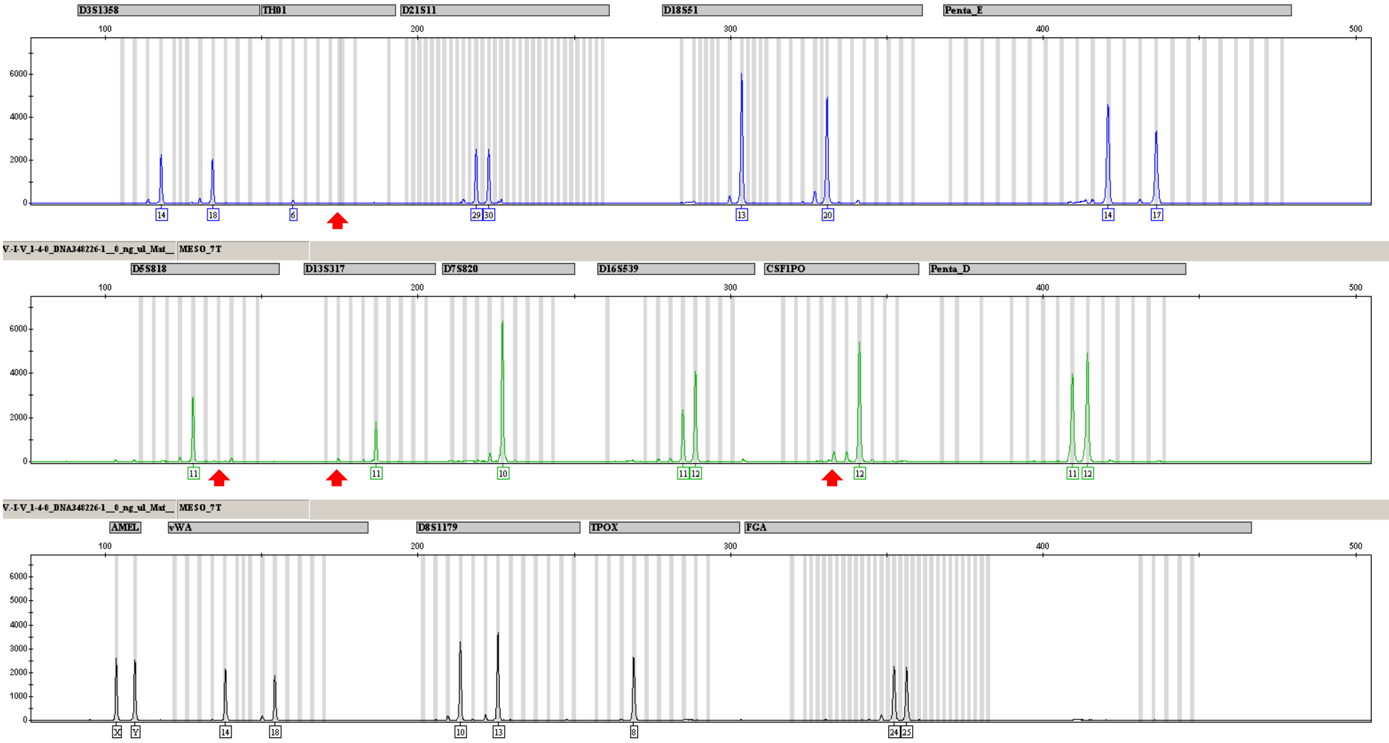

Supplementary Figure S5. An electropherogram of Powerplex PCR reaction. Top lines show STR profile in 7T tumor genomic DNA; no peak imbalance, indicative of a mixture in the sample, was noted. Bottom profile shows STR profile of the primary cell line MESO-7T. Arrows indicate positions of allele alterations compared to the parental genomic DNA.
