# Supplementary material for: Molecular profiling reveals primary mesothelioma cell lines recapitulate human disease
Source: Cell Death Differ. 2016 Feb 19;23(7):1152–64. doi: 10.1038/cdd.2015.165 (PMC4946883; doi:10.1038/cdd.2015.165)
Supplement: Supplementary Table 1 [file cdd2015165x6.pdf]

Cell Line ID	FCS Content in Media	Doubling Time (hours)
MESO-3T	2%	197.7641
MESO-3T	10%	141.5779
MESO-7T	2%	84.39534
MESO-7T	10%	62.49785
MESO-8T	2%	32.39441
MESO-8T	10%	38.62516
MESO-9T	2%	40.84242
MESO-9T	10%	41.87054
MESO-12T	2%	49.84425
MESO-12T	10%	37.13787
MESO-14T	2%	69.64033
MESO-14T	10%	66.99422
MESO-17T	2%	53.1163
MESO-17T	10%	40.84242
MESO-27T	2%	37.8541
MESO-27T	10%	31.62447

Supplementary Table 1. Doubling time of the primary mesothelioma cell lines.
