# Supplementary material for: Molecular profiling reveals primary mesothelioma cell lines recapitulate human disease
Source: Cell Death Differ. 2016 Feb 19;23(7):1152–64. doi: 10.1038/cdd.2015.165 (PMC4946883; doi:10.1038/cdd.2015.165)
Supplement: Supplementary Table 2 [file cdd2015165x7.pdf]

ABL1	KDR	BRAF	MPL	EGFR
EZH2	RB1	FLT3	SRC	HRAS
JAK3	APC	MET	CSF1R	NRAS
PTEN	FGFR2	SMARCB1	GNAQ	VHL
AKT1	KIT	CDH1	NOTCH1	ERBB2
FBXW7	RET	GNA11	STK11	IDH1
IDH2	ATM	MLH1	CTNNB1	PDGFRA
PTPN11	FGFR3	SMO	HNF1A	ERBB4
ALK	KRAS	CDKN2A	NPM1	JAK2
FGFR1	SMAD4	GNAS	TP53	PIK3CA

Supplementary Table 2. The Ion AmpliSeq™ Cancer Panel used in the TaqMan® Mutation Detection Assays to sequence gDNA from primary cell lines.
