# Supplementary material for: Molecular profiling reveals primary mesothelioma cell lines recapitulate human disease
Source: Cell Death Differ. 2016 Feb 19;23(7):1152–64. doi: 10.1038/cdd.2015.165 (PMC4946883; doi:10.1038/cdd.2015.165)
Supplement: Supplementary Table 3 [file cdd2015165x8.pdf]

Cancer Dataset	Common Genes (primary MM cell lines list, 500 genes)
Dataset: METABRIC (Breast cancer), subtype = "ER_IHC_status_pos"; 100 top genes associated (negative) with survival	32 genes:<br><br>CCNE2 FOXM1 TPX2 CENPN KIFC1 OIP5 KIF14 CDC20 PKMYT1 ASF1B EXO1 CEP55 TYMS E2F2 SGOL1 CENPF CDCA8 CDCA3 KIF23 RACGAP1 FBXO5 TTK PRC1 TICRR ZWINT KIF11 KIF4A CDCA5 CCNB2 UBE2C ESPL1 ORC6
Dataset: GSE30219 (Lung cancer), subtype = "ALL"; 300 top genes associated (negative) with survival	66 genes:<br><br>CSE1L CENPN FANCI KIFC1 MCM4 UBE2T TMPO MCM2 CDCA7 CDK1 PRIM1 CHAF1A TYMS SSRP1 MCM6 CENPF RRM1 RACGAP1 GMNN TTK MSH2 NEK2 KNTC1 MTFR2 KIF4A RAD51 CDCA5 CCNB2 ORC6 CCNE2 FOXM1 TPX2 ORC1 STIL ARL6IP6 OIP5 NEMP1 KIF14 FAM111B CDC20 BUB1B DKC1 EXO1 CEP55 MCM8 MCM3 POLQ CDT1 CDCA3 RAD54L MCM7 SAPCD2 PRC1 CKS1B SHMT2 MSH6 ZWINT PSMC3IP SNRPA NCAPD3 KIF18A RFC5 HNRNPH3 SLC25A19 UBE2C ESPL1

Supplementary Table 3. Top genes associated with survival from Lung and Breast cancer gene lists that are also present in Mesothelioma gene list (500 genes).
