# Supplementary material for: Molecular profiling reveals primary mesothelioma cell lines recapitulate human disease
Source: Cell Death Differ. 2016 Feb 19;23(7):1152–64. doi: 10.1038/cdd.2015.165 (PMC4946883; doi:10.1038/cdd.2015.165)
Supplement: Supplementary Table 4 [file cdd2015165x9.pdf]

#	P-value FDR (p-value)*	ODDS Ratio	Gene Expression Dataset	Number of top survival-associated genes in Primary MM cell line gene list (top 500 aberrant genes)
1	6.99e-44 (4.96e-46)	12.26	Dataset: GSE30219 (Lung cancer), subtype = "ALL"; 300 top genes associated (negative) with survival	66 (out of 500)
2	3.54e-24 (1.06e-25)	15.99	Dataset: METABRIC (Breast cancer), subtype = "ER_IHC_status_pos"; 100 top genes associated (negative) with survival	32 (out of 500)
3	1.70e-22 (6.72e-24)	7.30	Dataset: GSE4271 (Glioma (high-grade)), subtype = "Sex_F"; 300 top genes associated (negative) with survival	46 (out of 500)
4	2.83e-09 (4.31e-10)	3.04	Dataset: GSE39582 (Colon cancer), subtype = "Sex_M"; 700 top genes associated (positive) with survival	43 (out of 500)
5	2.73e-07 (4.71e-08)	6.52	Dataset: GSE16560 (Prostate Cancer), subtype = "fusion_0"; 100 top genes associated (negative) with survival	15 (out of 500)
6	0.0024 (0.000609)	4.07	Dataset: GSE32894 (Urothelial Carcinoma), subtype = "tumor_grade_G2"; 100 top genes associated (pos/neg) with survival	9 (out of 500)
7	0.0303 (0.0091)	3.14	Dataset: GSE13876 (Ovarian Cancer), subtype = "ALL"; 100 top genes associated (negative) with survival	7 (out of 500)

Supplementary Table 4. Full statistical details of the datasets showing the highest similarity with MM primary cell lines based on GO Term Enrichment Analysis.
